# Factors related to COVID-19 vaccine intention in Latino communities

**DOI:** 10.1371/journal.pone.0272627

**Published:** 2022-11-15

**Authors:** Adriana Perez, Julene K. Johnson, David X. Marquez, Sahru Keiser, Paula Martinez, Javier Guerrero, Thi Tran, Elena Portacolone

**Affiliations:** 1 Leonard David Institute of Health Economics, University of Pennsylvania School of Nursing, Philadelphia, Pennsylvania, United States of America; 2 Institute for Health & Aging, University of California San Francisco, San Francisco, California, United States of America; 3 College of Applied Health Sciences, University of Illinois at Chicago, Chicago, Illinois, United States of America; 4 Philip Lee Institute for Health Policy Studies, University of California San Francisco, San Francisco, California, United States of America; University of New Mexico Health Sciences Center, UNITED STATES

## Abstract

**Objective:**

To examine the effects of the COVID-19 pandemic among Latino communities, with an emphasis on understanding barriers and facilitators to vaccine intention prior to the development of the vaccine.

**Methods:**

Qualitative data were collected between April and June 2020 from 3 focus groups with Latino adults (n = 21) and interviews with administrators of community-based organizations serving Latino communities (n = 12) in urban (Los Angeles) and rural (Fresno) California, supplemented by Community Advisory Board input in May 2021to elucidate the findings. Data were analyzed with deductive content analysis.

**Results:**

We have identified four main themes that are barriers to vaccinating against COVID-19: 1) concerns about accessing appropriate healthcare services, 2) financial issues and 3) immigration matters, as well as 4) misinformation.

**Conclusions:**

Findings illustrate the pervasive role of addressable social determinants of health in the intention of rural and urban Latino communities in being vaccinated, which is a pressing public health issue. Policy implications: Findings provide evidence for a systemic shift to prioritize equitable access to COVID-19 vaccines to Latino communities.

## Introduction

The COVID-19 pandemic in the United States (US) has disproportionately affected racial and ethnic minoritized populations, further widening health disparities [1]. The largest US minority group, Latino individuals represent 18% (62 million) of the population yet account for 28% of COVID-19 cases [2]. Furthermore, Latino individuals are three times more likely to be hospitalized for COVID-19 [3] and have twice the risk for mortality from COVID-19 than non-Latino Whites [4].

Since the availability of COVID-19 vaccines in early 2021, the disparities between non-Latino whites and Latino have gradually diminished with Latino making up 16% (18 million) of people fully vaccinated versus 62% of whites as of 09/04/2021 [5]. However, most (58%) of the 42 million Latinos aged 18 and over are not fully vaccinated against COVID-19, which is a major public health issue, since the population is considered to be in the vaccination priority category due to highest risk for infection and severe death outcomes [6]. Furthermore, findings from a recent quantitative survey filled by 304 Latino respondents suggested that Latinos were less willing than whites to receive the COVID-19 vaccine [7].

To understand the barriers and facilitators to being vaccinated against COVID-19 among Latino, it is essential to elicit subjective perspectives from Latino individuals in both urban and rural US. As a result, the objective of this study was to identify the effects of the COVID-19 pandemic in urban and rural Latino communities, with specific attention to barriers and facilitators to the intention of being vaccinated for COVID-19.

## Method

### Study design

We used qualitative methods involving focus groups, semi-structured interviews, and content analysis to examine the effects of the pandemic, with specific attention to the intention to receive the COVID-19 vaccine. Focus groups (FGs) with Latino adults were conducted to foster exchange of perspectives and to gain collective narratives, which facilitates participants’ empowerment [8]. Drawing from prior research [9], we also conducted one-on-one semi-structured interviews with administrators of community-based organizations serving primarily Latino communities to elicit their insights about their served communities. Finally, one year later, after vaccines were available, we elicited the insights of members of our Community Advisory Board (CAB) of Latino leaders to validate and expand our findings.

### Recruitment

Participants were recruited from an ongoing qualitative parent study in California that began in 2019 about factors influencing Latino participation in research [10, 11]. Inclusion criterion for in-depth interview participants was working as administrators of organizations serving primarily Latino individuals (e.g., farmworkers, immigrants). Inclusion criteria for FG participants included being older than 18 years of age and self-reporting as Latino/Hispanic.

Between April and June 2020, we used two strategies to recruit participants in this study. First, Latino adults who already participated in the first FG of the parent study were invited to attend a second FG for this investigation on the effects of the pandemic. To originally recruit Latino adults in our parent study, we sought the support of local CBOs administrators. Specifically, CBO administrators solicited participation by disseminating our recruitment flyers showing university logos and the name and address of their CBO. Some CBO administrators also hosted presentations of our team. Second, we recruited administrators of CBOs located in Fresno and Los Angeles that were indicated as trusted in the first round of FGs of the parent study. Specifically, after we identified trusted CBOs, we asked their key administrators if they were available to be interviewed for about one hour. The study was approved by University of California San Francisco (UCSF) Committee on Human Research (#17–23278).

We also involved our existing CAB, which included four women and two men; all were Latinos; three were from the San Francisco Bay Area, two from Fresno, and one from Arizona. Professional backgrounds of the CAB members included: leaders of non-profit organizations, leaders of advocacy organizations focused on dementia, administrators of dementia services as well as housing services.

### Data collection

Data were collected from 12 interviews and 3 FGs (April-June 2020) and 1 CAB meeting (May 2021). The study took place in rural (Fresno) and urban (Los Angeles) sites in California. Interviews and FGs were led by Latino researchers over videoconferencing (Zoom), audio recorded with professional audio-recorders, and professionally transcribed. Informed consent was obtained before collecting data. FG participants were asked about their personal attitudes towards being tested for COVID-19 and being vaccinated once vaccines became available, as well as perceptions of Latino communities on these topics. FGs lasted 90 minutes. During interviews, participants were first asked about the effects of the pandemic on Latino communities. Next, interviewees were asked about barriers/facilitators to COVID-19 testing and vaccinations. Interviews lasted one hour. All FGs and interviews were conducted in English, except one interview conducted in Spanish. Researchers audio recorded fieldnotes including reflections and descriptions right after each FG or interview. All participants received a $40 gift card. Later, after vaccines were publicly available, the results of the study were presented to the six-member CAB by the lead author during videoconferencing to solicit feedback. We also elicited new insights because the CAB convened after COVID-19 vaccines were available. Specifically, during one-hour meeting, the first author presented the results of the study over videoconference to seek agreements, disagreements, or expansions. To thank them for their effort, each CAB member received $100 honorarium.

### Data analysis

Transcripts of interviews, FGs, fieldnotes, and the CAB meeting were entered into Atlas.ti, Version 8 software for data analysis and analyzed with deductive qualitative content analysis [12, 13], a commonly used method of qualitative data analysis [14–16]. Because the study was exploratory, we did not draw from any theoretical framework. Initially transcripts were analyzed line by line by the last author to identify specific effects of the pandemic in Latino communities. Specifically, she attended to quotes about barriers and facilitators to COVID-19 test and vaccine intention. A code was created every time a particular barriers and facilitators was identified. A senior coder (Thi Tran) reviewed her codes until interpretative convergence was achieved. Definitions of codes and related categories were documented in a codebook and shared with the research team. Next, other independent coders coded the rest of the transcripts; each coded transcript was reviewed by a coder to achieve interpretative convergence. Additional codes were added with the approval of the research team. Saturated themes were then identified by the last author through making connections among codes, writing memos, and having iterative discussions with the research team. Afterwards, we compared and contrasted narratives from FGs and interview participants, as well as CAB members to assess whether they corroborated, negated, or expanded on one another, which adds depth and rigor to the analysis.

## Results

### Demographic characteristics of participants

Participants’ characteristics are detailed in Table 1.

**Table 1:** Demographic characteristics of participants.

Characteristics	*n* (percentage)		
	Focus Group (n = 21)	CBO Admin (n = 12)	Total (n = 33)
Age (mean, SD^a^, range)	26 (SD = 11) (19–55)	43 (SD = 9.8) (29–58)	30 (SD = 11.7) (19–58)
Sex, Female	13 (62%)	8 (67%)	21 (64%)
Race			
White	5 (24%)	4 (33%)	9 (27%)
American Indian or Alaskan Native	2 (10%)	1 (8%)	3 (9%)
More than one race	3 (%)	0	3 (9%)
Not provided/other	11 (%)	7 (58%)	18 (55%)
High school education or above (%)	18 (86%)	11 (92%)	29 (88%)
Location			
Rural, Fresno	13 (62%)	9 (75%)	22 (67%)
Urban, Los Angeles	8 (38%)	3 (25%)	11 (33%)
Heritage			
Mexico	18 (86%)	12 (100%)	23 (70%)
Central America (El Salvador)	2 (9%)	0	2 (6%)
Not provided	1 (5%)	0	8 (24%)

^a^SD = standard deviation

### Central themes

The main theme to COVID-19 vaccination identified and unanimously validated by CAB members, was a tension between the intention towards COVID-19 testing and vaccination and four barriers: difficulty in accessing appropriate healthcare services, financial and immigration matters, respectively, and misinformation. These themes and examples of participant quotes are described in Table 2.

**Table 2:** Themes and examples.

Theme	Quote
**Limited access to health care services**	Healthcare access is a big concern that the coronavirus has exacerbated, because as you know, here in the Central Valley, we have a lot of uninsured or underinsured low wage Latinos from various immigration backgrounds. So that’s kind of some of the predominant concerns that I’ve heard from the community. *Administrator*
	Many of them don’t have healthcare, so they will wait until they absolutely have to go to the doctor, even if they’re really sick, in order for them to access those services because of the lack of funds at home and having to have to decide between getting medical help or putting food on the table. *Administrator*
	It’s easier when the vaccine can come to the properties, so like… “Oh, I have to go out of my way and take transportation and then wait in line”, versus “I can just go to the courtyard of my home and get it done, and then total wait time is like 30 minutes.” *CAB member*
**Financial concerns**	You can’t tell a broke Hispanic what to do because he’s struggling. *FG participant*
	Latinos that are working that are not working now, and I think like I said, the uncertainty of when they can get back to work and not having money now to pay the bills is a big one. *Administrator*
	We know that right now the number one need when it comes to our farmworker relief fund that we have for farmworkers is rent relief. *Administrator*
	Probably the vaccine is free but what about the treatment? What if you get tested positive and you need something that’s not covered by your health insurance? And that fear that it’s going to be something that you can’t afford, it’s probably maybe you don’t want to find out if you’re sick or not. *FG participant*
	There’s no guarantee that there’s a safety net to catch you if you do test positive. So it’s almost like you will be punished, in a way, if you test positive, not directly but in other ways. So I think that’s a big deterrent. *FG participant*
**Immigration matters**	Basically all roads lead to the older Hispanic culture. All roads lead to the possible deportation. … Anything that could possibly lead to deportation, there’s an extreme apprehension. So being forced to take the test or being forced to take the immunization or whatever, to be put on a list and possibly be deported, that’s always a concern in the back of the older Latino population especially. *FG participant*
	If I show up and have a positive test, will they ask more questions about my citizenship? *FG participant*
	As of February 24 [2020], a new public charge policy was passed that now says if the person has ever received programs like housing assistance, full scope Medi-Cal, food stamps and long term care at a hospital, then those individuals may be unable to get a green card because of the receipt of those benefits for a certain amount of time. But in reality, a lot of undocumented immigrants aren’t eligible for those kinds of benefits. However, there’s a lot of individuals who do receive certain safety net programs like emergency Medi-Cal, and they fear that those benefits may be used against them. *Administrator*
	If it [vaccine] is counted as a public charge, a lot of folks are going to not want to access it for fear of having that on their record. *Administrator*
	The fear that the immigrant community living in this country has because of the statements that the president [Trump] has been making. We know that he makes a lot of statements, so after he makes the statements, many people are afraid of going to the places that are related to the government. I’m talking about clinics, hospitals. Why? Because they have heard people saying, “Someone went to court,” for example, “and immigration detained him there and deported him.” So many people have that fear. “I don’t know, maybe I shouldn’t go out, maybe I won’t go because it is related to the government.” *Administrator*
**Limited information**	I feel like the older generations just lack the information on it [pandemic]. They do have a phone, but I feel like they don’t know how to research. […]Well, my dad, my grandfather, my uncles, they really don’t know how to use the internet or any of the sites, the CBC, the WHO. So I feel like we have more information available to us than they have. So they just close themselves in their own bubble and listen to Telemundo, to Univision, right? And of course the media’s going to manipulate the information that is given to them. So I feel like that’s why they’re going to be more closed to it while we have more an open mind to it. * FG participant*
	I think that through education it could really help show people that there are positives in it because a lot of people still today don’t take the flu vaccine either who are part of the Latino and Chicanx culture and society. And communities don’t really understand how important it is because they’re being told different things throughout our community. They’re always shown the negative parts of it. *FG participant*
	What would definitely motivate the community would be facts. As I said, with all the uncertainty that’s going on, truth would be a huge thing. […]. So of course if we show other stories and we express how other likeminded people are, I feel like that would lure a lot of people in for sure. Because family is important and if that gets out there, as well as like facts and truth, I feel a lot of people would be more likely to want to take it [vaccine], to be motivated. *FG participant*
	So I think more education has to be done for learning that. Because even though we are in our houses and we’re trying to stay away from it, we don’t really understand it. And when we think we’re trying or we’re like getting there, something new comes up and we are lost again. *FG participant*
	Just really being intentional on creating the messaging [about the COVID-19 vaccine] so that when it gets to the Latino families in the community, there’s just a lot of information for them. And create a space that makes it safe for them to ask questions. *Administrator*

#### Theme 1: Limited access to healthcare services

A major concern was limited access to healthcare services, which influenced the intention to being tested or vaccinated. Three factors limited access. First, participants pointed out that Latino individuals are often uninsured or underinsured because of immigration status and employment with limited benefits. The risk of losing employment because of the pandemic would also result in losing healthcare insurance. One administrator mourned the deaths of Latino individuals who did not seek medical assistance when experiencing COVID-19 symptoms because they could not “count on health insurance.” Administrators explained that a consequence of having limited/no insurance is that most healthcare visits occur in emergency rooms, which are associated with higher costs. Related, the costs of getting medical services were burdensome to the point one had “to decide between getting medical help or putting food on the table.”

A second factor was that the public healthcare systems were already strained before the pandemic, with scarce healthcare providers and long wait times, especially in rural settings. One administrator explained that, because of the pandemic, she closed the mobile health clinics in rural areas, an essential resource for rural Latino communities, to protect her employees.

A third limiting factor was a reluctance to interact with healthcare providers. Providers were often perceived as government agents. One administrator pointed to the sense of unsafety related to “going to the doctor.” Another administrator explained that fears triggered by the former president’s anti-immigration policies contributed to fears of dealing with government-agency-like figures such as healthcare providers. These fears were fueled by accounts of Latino individuals being taken to court, detained, or deported following interactions with “clinics and hospitals related to the government.” Healthcare institutions were avoided because of fear of contracting COVID-19. A distrust towards Western medicine was also noted, especially considering the reliance of some Latino communities on traditional herbal remedies.

Questions were raised about the future availability and costs of COVID-19 tests and vaccinations for populations who are underinsured, as well as the availability of testing and vaccination sites in rural areas. Concerns were also raised about not having adequate healthcare for side-effects of vaccinations. On a positive note, most participants said that Latino communities would consider being tested or vaccinated if these were easily accessible and at no cost. CAB members confirmed these projections when noting that Latino communities were vaccinated because of no-cost vaccinations and free transportation. CAB members underscored the importance of “intentional” approaches that make it easy to get vaccinated, such as support with registration and having vaccination sites available in multiple locations close to one’s home. They suggested the idea of offering a bundle of free services (e.g., blood pressure, eyesight checks) at vaccination sites.

#### Theme 2: Financial concerns

The financial cost of the pandemic to Latino communities was at the forefront of all who were interviewed. Major concerns stemmed from lost earnings due to sheltering-in-place policies, the unknown duration of sheltering-in-place, job losses, as well as difficulties with paying for food, utilities, and housing for the family units. For some, financial insecurity was exacerbated by being ineligible for unemployment support and coronavirus relief funds due to immigration status, as detailed in the next section. Having a post office box address, which is common in rural areas, disqualified families from receiving government pandemic relief funds.

The importance of financial support for paying rent or avoiding evictions was often stressed. The need to work despite the pandemic increased the risk of infecting oneself or their family. Also, the type of employment often increased this risk because many Latino individuals worked as essential workers (e.g., agricultural fieldwork, packing houses, warehouses). Concerns were raised about unsafe working conditions, ranging from commuting in “crammed vans” to not receiving PPEs from employers. Living in multigenerational households and highly populated areas also increased the risk of being infected, CAB members added. Administrators explained that because of poverty, some Latino individuals may have to face the dilemma of whether “to buy groceries or Clorox wipes, gloves, or masks.”

Concerns were also raised about the potential costs of being vaccinated, which would include transportation, the vaccine (at the time of the study, it was unknown that vaccinations would be no cost), as well as potentially lost earnings for being unable to work due to vaccination side-effects. Participants underscored the importance of offering financial support to those unable to work because of a positive test result or the side-effects of the vaccine, as well as reimbursements for travel and no-cost testing and vaccinations. Finally, the need to work was mentioned as an incentive to be vaccinated.

#### Theme 3: Immigration matters

Concerns about immigration status uniformly emerged. “All roads lead to the possible deportation,” one FG participant noted. An administrator explained that immigration concerns are embedded in Latino communities because it is common for families to have one spouse undocumented and other members with US citizenship.

Administrators also explained that concerns related to immigration peaked following the passing of a law that narrowed the criteria to receive a green card under the assumption that potential US citizens must provide proof of self-sufficiency and not being a “public charge.” Thus, starting February 24, 2020, the list of benefits considered as “public charges” expanded to include food stamps, most forms of Medicaid, public housing, and hospitalization [17, 18]. Specifically, concerns were raised on whether being vaccinated could be defined as a public charge, thus eliminating green card eligibility. Conversely, concerns were raised on the legal consequences for not being vaccinated. COVID-19 tests were also approached with caution for fear that a positive test could taint one’s immigration status in unpredictable ways. On a positive note, some administrators reflected that if it was made clear that vaccination and testing did not affect immigration status, Latino communities would be inclined to take advantage of both.

#### Theme 4: Limited reliable information

Concerns were raised about limited reliable and positive information about the pandemic. Participants stressed the confusion, uncertainty, and negativity projected by the media and amplified by Latino individual’s inclination to remain reserved about private matters. CAB members were concerned about pervasive messages in the social media (e.g., Facebook, YouTube) dissuading Latino communities from getting vaccinated. Some FG participants were particularly concerned about the misinformation of older Latino cohorts who “closed themselves in their own bubble” because of sensationalistic Spanish news media. Concerns were raised about Latino individuals with low literacy who are vulnerable to misinformation for whom pandemics and vaccinations are “new and unknown” matters difficult to comprehend. One administrator was distressed about the limited awareness of Latino fieldworkers about the importance of wearing PPEs. Another stressed that some indigenous Latino communities (e.g., Oaxaqueño) speak neither English nor Spanish and are cut off from traditional “social networks.” Similarly, CAB members underlined the importance to consider the diversity of Latino communities when disseminating information.

The critical role of young people who could access information from reliable sources (e.g., CDC, WHO) via their smartphones emerged. To address misinformation and related fears, most participants pointed to the importance of increasing the education of Latino communities about vaccinations and COVID-19 in general with “facts” and “science.” Specifically, participants recommended that reliable information should come from trusted sources (local organizations, promotoras de salud, key community members, celebrities) using culturally relevant methods (e.g., playing Spanish music). Indeed, the pivotal role of trusted sources was stressed by the CAB. Spanish television programs were also indicated as important platforms to reach Latino communities; while Spanish radio stations were critical to reach rural communities who often listened to the radio while working. A motivator to increase vaccinations was stressing the ability of safely interacting with family members and showing positive accounts of acquaintances getting vaccinated. Another lever was to clearly explain the consequences of being vaccinated regarding immigration matters, especially with the definition of “public charge.”

## Discussion

In this study we report on barriers and facilitators that may impact vaccine COVID-19 uptake in Latino rural and urban communities in California. Perspectives were elicited prior to vaccine distribution, at a time when most Latino individuals were deeply concerned about their place in US society [19]. Identified themes included limited access to healthcare services and reliable sources of information, plus financial and immigration concerns. Themes point to the marked role of social determinants of health that exacerbate health disparities among Latino communities amid the pandemic [20]. This is one of the first qualitative studies focusing on barriers and facilitators to COVID vaccine uptake in both urban and rural Latino communities in the US, which is an important contribution to decreasing COVID-19 health inequities.

COVID-19 testing and vaccine uptake were deterred by limited access to healthcare services characterized by limited/no insurance coverage, a strained public health care system, and avoidance of healthcare providers. This finding is consistent with studies that emphasize limited access to healthcare services during the COVID-19 pandemic among monolingual Spanish Latino individuals [21, 22]. While the reluctance of Latino individuals to access healthcare providers, due to language barriers and mistrust has been documented [21], our study adds that the perception of healthcare providers as government representatives increases mistrust. Also, to address vaccine outreach efforts for Latino communities, and confirmed in the literature [23, 24] is a preference for herbal remedies.

Financial concerns also tempered the intention to being vaccinated and tested against COVID-19. Prevalent were concerns of losing earnings from precarious employment to be tested or vaccinated, experiencing post-vaccination symptoms, or travelling to testing or vaccination sites, or the potential cost of testing and vaccination services. The few studies that focused on the impact of COVID-19 on Latino communities consistently found that these communities reported the financial constraints that threaten employment and immigration status especially if they tested positive [21, 24, 25]. Since many Latino individuals work in occupations considered essential and are likely to work in conditions with limited access to proper PPE and limited social distancing [26, 27], their risk for COVID-19 further contributes to financial concerns.

Immigration concerns also deterred Latino communities from considering being vaccinated/tested. Although this study included urban and rural Latino communities in California mostly from Mexico, similar findings were also reported in the state of Virginia among Latino communities from other heritages [26]. Latino immigrants’ avoidance of public benefit programs was known before the pandemic [28], however a study [29] found that the avoidance of public resources among Latino immigrant families intensified after the 2016 presidential election.

Limited access to reliable sources of information was yet another barrier to consider being vaccinated/tested. This theme emerged in other studies of among Latino communities through the pandemic. According to a study conducted in Texas, [30] language barriers contribute to misunderstandings and mistrust of government officials. Another study [21] found that Latino communities in the Midwest reported confusing messages from social media. Further, similar findings [31] were reported during the H1N1 influenza pandemic among Latino communities, including conspiracy theories. One important contribution of this study is the insight that Latino communities view their healthcare providers as a trusted source of information. Furthermore, our findings suggest that sub-cultural differences and generational differences may exist in how COVID-19 information can be effectively tailored and communicated, with younger cohorts supporting older ones to understand events.

Limitations included the small sample based in one state. While most findings are supported with studies conducted in other regions among diverse Latino cultural heritages, it is not possible to generalize to the entire US Latino population. Furthermore, data were collected prior to vaccine distribution and before efforts were implemented to increase vaccinations in Latino communities. Also, this study did not recruit monolingual Spanish speakers; future studies should recruit them because they often reside in counties with higher COVID-19 cases [32].

## Public health implications

Our findings point to the critical need for robust, culturally, and linguistically relevant strategies supported by public policies to achieve equitable vaccine distribution among Latino communities. Consideration should be given to the unique needs of those living in rural settings, especially farmworkers [33]. Overall, we have five specific recommendations. First, more coordinated efforts between federal and state government are needed to ensure clear messaging regarding the availability of vaccine services, regardless of immigration documentation status. Second, there is a need for pointed communication strategies. Public service announcements must directly address the concerns of Latino communities regarding immigration status, health insurance and the availability of “no cost” vaccines. Third, to reach rural Latino communities, more resources are needed to support COVID-19 vaccination efforts from bilingual, trusted health care provider clinics, local Spanish-speaking organizations (e.g., local churches, immigration centers, farmworkers organizations), and essential occupation sites with the highest Latino workers identified (e.g., packing houses). Fourth, as also recently underlined by Liebman et al in this journal [33], to support rural Latino communities, it is important to foster partnerships between clinical providers, employers of farmworkers, and advocacy organizations under the aegis of state and federal public health departments. Fifth, the CAB recommendation for bundling vaccinations with other free wellness services is an excellent strategy for any organization serving as a vaccination hub. And sixth, policies that address underlying structural barriers are needed. Every worker in the U.S. should benefit from federal COVID-19 relief programs, including paid sick leave and family leave.

To conclude, prior to the pandemic, the Latino population, 62.1 million in the US, already experienced health inequities largely driven by social determinants of health. As discussed in this paper, the COVID-19 pandemic has only deepened and put a spotlight on these well-known inequities. As Corbie-Smith has suggested [34], it is time for a systemic “shift that demonstrates trustworthiness and commitment to equity.” Our findings provide rigorous evidence for this shift because they illustrate the pervasive role of addressable social determinants of health in the intention of rural and urban Latino communities in being vaccinated, which is a pressing public health issue.

## Supporting information

S1 File (DOCX)
